# Quittr: The Design of a Video Game to Support Smoking Cessation

**DOI:** 10.2196/games.6258

**Published:** 2016-12-01

**Authors:** Ivan Bindoff, Kristy de Salas, Gregory Peterson, Tristan Ling, Ian Lewis, Lindsay Wells, Peter Gee, Stuart G Ferguson

**Affiliations:** ^1^ University of Tasmania Hobart Australia

**Keywords:** smoking cessation, video games, mobile phone, motivation

## Abstract

**Background:**

Smoking is recognized as the largest, single, preventable cause of death and disease in the developed world. While the majority of smokers report wanting to quit, and many try each year, smokers find it difficult to maintain long-term abstinence. Behavioral support, such as education, advice, goal-setting, and encouragement, is known to be beneficial in improving the likelihood of succeeding in a quit attempt, but it remains difficult to effectively deliver this behavioral support and keep the patient engaged with the process for a sufficient duration. In an attempt to solve this, there have been numerous mobile apps developed, yet engagement and retention have remained key challenges that limit the potential effectiveness of these interventions. Video games have been clearly linked with the effective delivery of health interventions, due to their capacity to increase motivation and engagement of players.

**Objective:**

The objective of this study is to describe the design and development of a smartphone app that is theory-driven, and which incorporates gaming characteristics in order to promote engagement with content, and thereby help smokers to quit.

**Methods:**

Game design and development was informed by a taxonomy of motivational affordances for meaningful gamified and persuasive technologies. This taxonomy describes a set of design components that is grounded in well-established psychological theories on motivation.

**Results:**

This paper reports on the design and development process of Quittr, a mobile app, describing how game design principles, game mechanics, and game elements can be used to embed education and support content, such that the app actually requires the user to access and engage with relevant educational content. The next stage of this research is to conduct a randomized controlled trial to determine whether the additional incentivization game features offer any value in terms of the key metrics of engagement–how much content users are consuming, how many days users are persisting with using the app, and what proportion of users successfully abstain from smoking for 28 days, based on user-reported data and verified against a biochemical baseline using cotinine tests.

**Conclusions:**

We describe a novel, and theoretically-informed mobile app design approach that has a broad range of potential applications. By using the virtual currency approach, we remove the need for the game to comprehensively integrate the healthy activity as part of its actual play mechanics. This opens up the potential for a wide variety of health problems to be tackled through games where no obvious play mechanic presents itself. The implications of this app are that similar approaches may be of benefit in areas such as managing chronic conditions (diabetes, heart disease, etc), treating substance abuse (alcohol, illicit drugs, etc), diet and exercise, eating disorders (anorexia, bulimia, and binge eating), and various phobias.

## Introduction

### Smoking and Cessation

Smoking is recognized as the largest, single, preventable cause of death and disease in the developed world. It is associated with an increased risk of heart disease, stroke, cancer, emphysema, bronchitis, asthma, renal disease, and eye disease. While the majority of smokers report wanting to quit, and many try each year, smokers find it difficult to maintain long-term abstinence [1-3].

The delivery of education and support to smokers has been the backbone of smoking cessation programs for decades. Such programs were traditionally delivered in face-to-face sessions with trained tobacco cessation counselors, or via self-help booklets and websites, but more recently researchers have turned to technological solutions such as mobile phone apps in order to deliver content to smokers in a way that can be easily scaled up to population levels [4-6]. Regardless of the delivery modality, behavioral support programs have been found to be mildly effective [7], with the educational content involved in such programs (including advice on when and how to use pharmacological support) appearing to have the greatest impact on cessation rates.

Despite significant investment in the development of such programs, a persistent issue is that smokers do not readily engage with the cessation content delivered. Failure to engage with the “active content” of cessation programs is problematic as it limits the potential effectiveness of such programs: the education and support delivered cannot hope to be effective if it is not used by smokers during their quit attempt. Even if users engage initially, it is difficult to keep them interested beyond the first few days (retention). Identifying mechanisms that encourage smokers to engage with smoking cessation content requires ongoing investigation.

### Video Games and Health Interventions

Video games are immensely popular, with recent statistics indicating that in the United States 63% of households are home to at least 1 person who plays video games regularly (3 hours or more per week) and 65% of households own a device used to play video games [8]. Rather than video games being seen as simply time-wasting entertainments, they are now recognized as an integral part of our modern society, with games and play increasingly informing other domains of our everyday life.

Research on video games now shows that games have the ability to keep people engaged for a long time, build relationships and communities among players, and cultivate their creative potential. Furthermore, games have been shown to have the power to support knowledge acquisition and even to bring about behavior change [9-11].

Games with a purpose, also known as “serious games,” look much like traditional entertainment games, but differ in that they have a defined purpose, outcome, or message that the creators of the game are trying to get across to a particular audience [12]. By employing all the fun and engaging elements found in games and applying them to real-world or productive activities, they have the potential to influence behavior or improve engagement in tasks not generally considered “fun,” such as learning, exercising, and practicing.

Within the health domain there have been a wide range of successful serious games initiatives trialed. A 2008 review of 27 publications reporting on 25 video games that promoted health-related behavior change, identified that most of the studies demonstrated positive health-related changes from playing the video games [13].

More recently, a 2012 review included 38 publications regarding video games that provide physical therapy, psychological therapy, improved disease self-management, health education, distraction from discomfort, increased physical activity, and skills training for clinicians [14]. Among the 38 studies, a total of 195 health outcomes were examined. Results indicated that video games improved 69% of psychological therapy outcomes, 59% of physical therapy outcomes, 50% of physical activity outcomes, 46% of clinician skills outcomes, 42% of health education outcomes, 42% of pain distraction outcomes, and 37% of disease self-management outcomes. Further, in 2014, a meta-analysis of 54 serious digital game studies for healthy lifestyle promotion reviewed the overall effectiveness of serious digital games on healthy lifestyle [15], and demonstrated small positive effects on healthy lifestyle promotion outcomes.

### Video Games and Smoking Cessation

The concept of using games to promote smoking cessation is not entirely new. There is some existing work that explores the potential of the idea, and even some existing game-based interventions have been developed. Most early attempts at incorporating video game elements have focused on the delivery of a distraction, to help the smoker get through an episode of craving. Crave-Out was an early attempt at this style of game, producing a pattern matching memory game and using the Questionnaire of Smoking Urge-Brief measurement before and after play to determine if it helped to reduce cravings. A small but insignificant reduction in cravings was measured. Critically however, the authors determined during pilot testing that negative reinforcement had a deleterious effect on smokers, as it reminded them of smoking, and this finding is worth bearing in mind in future designs [16]. Subsequently, similar “distraction minigames” have also been incorporated into other mHealth-based smoking cessation apps such as SmokeFree 28 [5].

The most grass-roots evidence to support the idea of game-based smoking cessation interventions comes from Raiff et al [17], who explored the link between video game usage and smoking. They determined that 74.5% of smokers play video games (more than nonsmokers), and that 63.7% of participants believed that a video game–based intervention would motivate smokers to quit [17]. There is some speculation that the “addictive tendencies” of smokers makes them more likely to become frequent video game players.

Hall et al [18] tried to qualitatively determine, from expert opinion, key design characteristics for a successful video game–based smoking cessation intervention. After interviewing experts in game development, tobacco, and health behavior a number of themes emerged. In particular, it was noted that targeting youths through games was likely to be an effective strategy that extensive iterative prototyping was required to create a compelling game experience, and that incorporating behavioral change techniques was likely to result in improved outcomes.

There is also some preliminary evidence exploring Internet implementations. QuitIT is an Internet-based video game that is designed to help smokers practice coping strategies for cravings. Initial evaluation suggests the game was useful in teaching strategies for coping with urges [19], although there is not yet any data to suggest that it improves quit success rates.

Traditionally, serious games in the health behavior change space have focused on directly encouraging the behavior of interest (eg, promoting physical activity by requiring movement as part of the game play). An alternate possible use of games–one that we explore here–is that games could be embedded with education and support content, such that the game play simply acts as the hook that compels the user to access and engage with relevant educational content, in exchange for incentives that further the players goals in the game. Such game play may encourage engagement with proven educationally based intervention strategies, and thereby improve health outcomes.

It has been suggested that games are fun because they appeal to some core drives that motivate us toward certain activities, such as meaning, empowerment, social influence, unpredictability, avoidance, scarcity, ownership, and accomplishment [15]. If we can identify the elements of games that support the acquisition of smoking cessation content, and build a game that provides an engaging environment for players to remain engaged with such content, we may be able to positively influence smoking cessation outcomes.

### Theoretical Basis for Design

This focus aligns with the assessment that reflective and automatic motivation toward smoking cessation can be enhanced by interventions, such as education, persuasion, intensification, coercion, and modelling [20]. However, the actual content component of the app itself is flexible and has been designed to be easily updatable as new content is developed.

### Game Mechanic and Game Elements Design Phase

**Figure 1:**
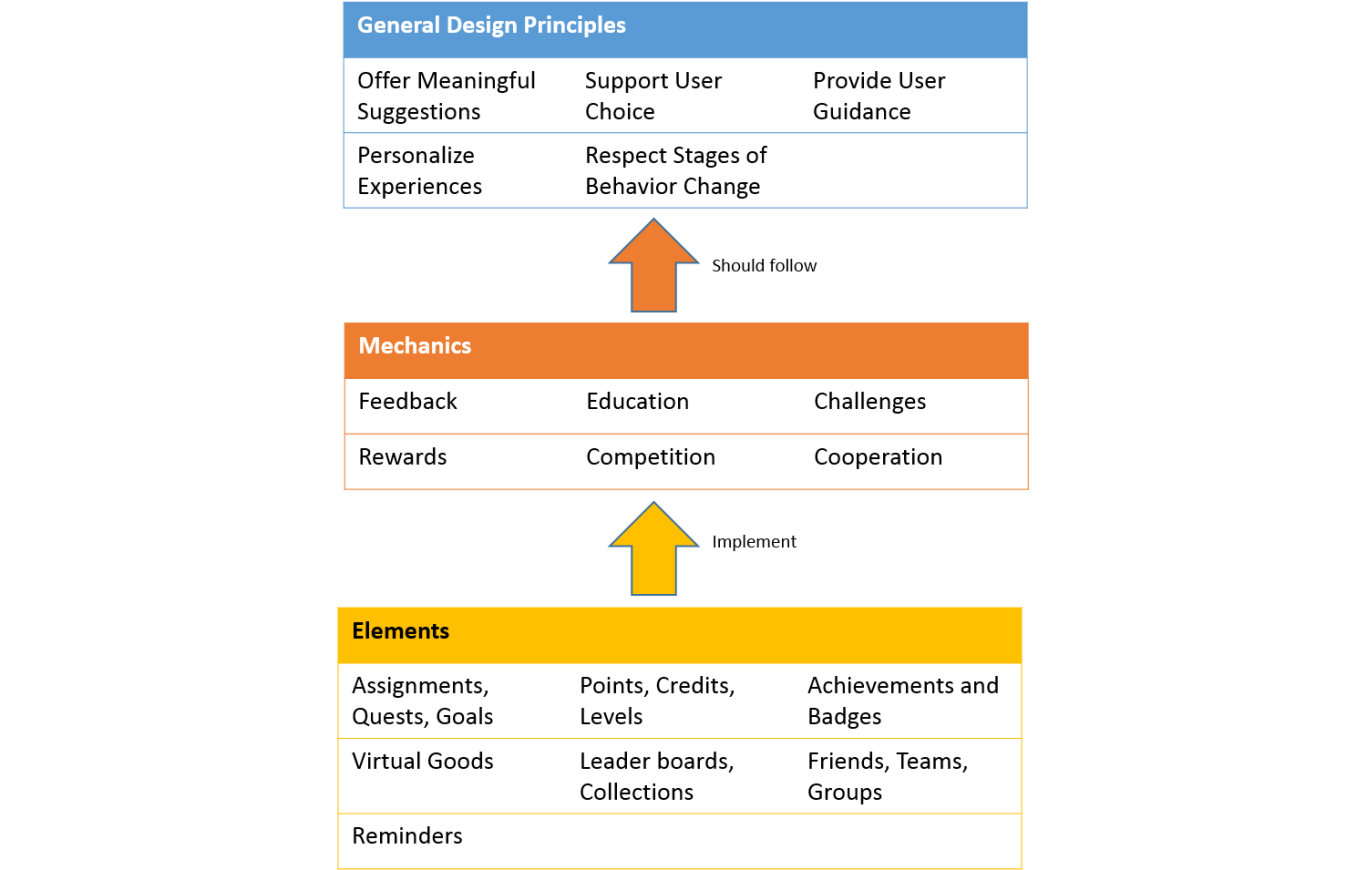
Design principles and mechanics for persuasive game design [17].

Within the cycle of design and development, the initial Game Mechanic and Elements design phase was informed by a taxonomy of motivational affordances for meaningful gamified and persuasive technologies (Figure 1) [20]. This taxonomy describes a set of design components that is grounded in well-established psychological theories on motivation [20].

To allow for meaningful gamified and persuasive systems, designers should choose components depending on both the objectives and the users of the system [20]. To guide this activity, the taxonomy outlines both general design principles and mechanics that game designers might benefit from considering, and that might assist in ensuring theoretically grounded serious game development, as is required in this intervention.

## Methods

### Overview of the Design

Based on the theoretical underpinnings described above, we designed and developed a mobile phone game called Quittr to support people in their attempt to quit smoking. This design borrowed many elements from existing literature [21,22] and examples of mHealth smoking cessation apps, most notably SmokeFree 28 [5]. The focus was on creating a platform that would enable the delivery of proven effective smoking cessation support content, but with game elements integrated throughout to improve user engagement and retention.

**Figure 2:**
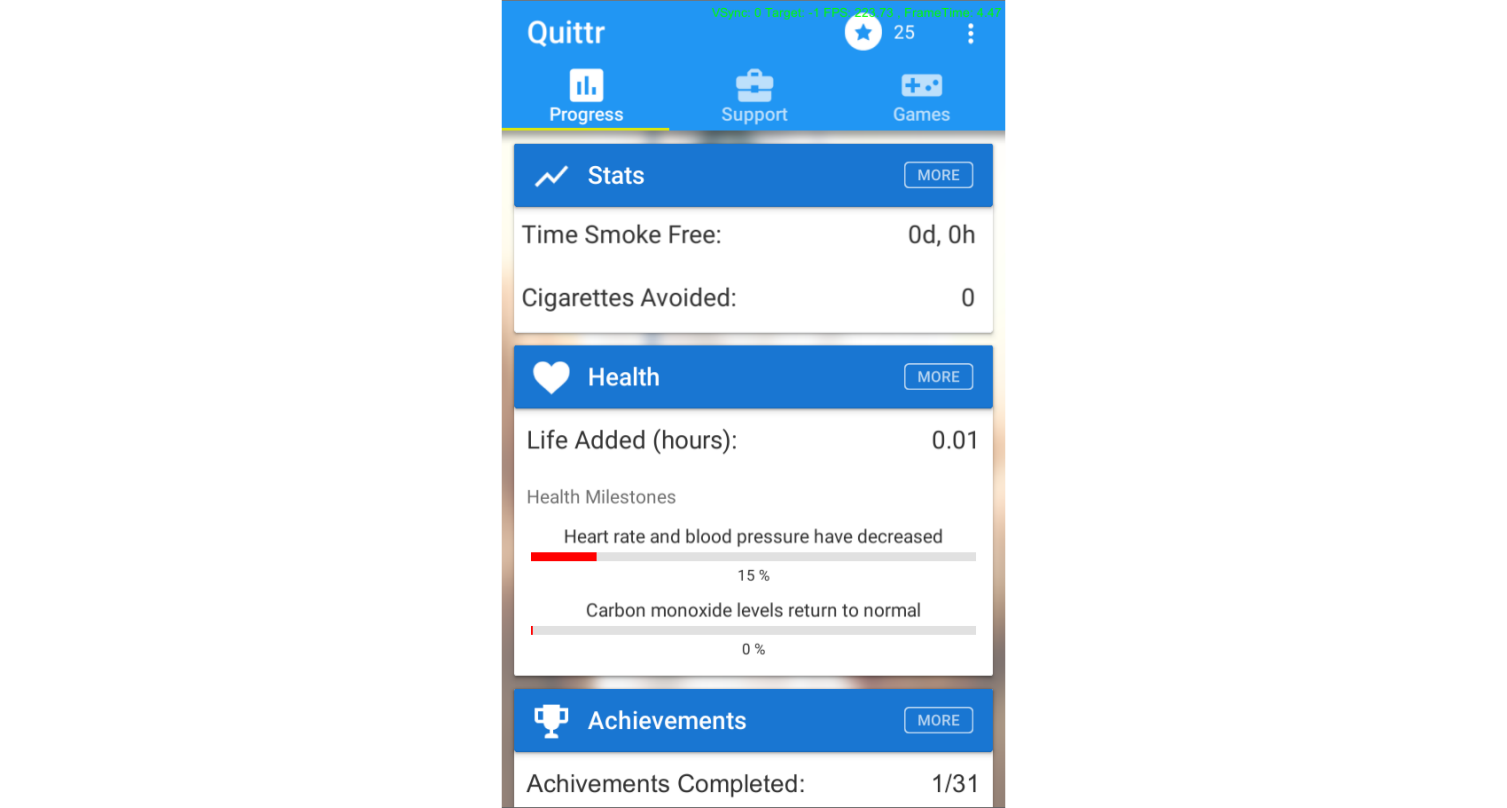
The main dashboard of Quittr.

An app was produced compatible with both Android and iOS mobile phones and tablets. Upon opening the app, users are greeted with a dashboard system separated into 3 main pages, which they can swipe between (Figure 2), styled in adherence with the popular Google Material user interface design guidelines [23].

### Data Collection

When the user first opens the app they are prompted to complete an entry survey, which includes questions about the user’s smoking status, demographics, health, and financial goals, as well as questions about the user’s intentions in using the app. On subsequent openings, the user is asked if they have smoked any cigarettes that haven’t been entered into the app. If the user indicates yes to this question, they are asked to indicate how many cigarettes they smoked on each day that has elapsed since their last log. If the user has not opened Quittr for 12 hours they are prompted with a notification on their device asking them to come back and log their cigarettes. This will repeat daily for several days if they still fail to check-in.

At the end of 28 days or when the user indicates their current quit attempt has failed, they are also asked to complete an exit survey. This asks questions about their opinions of the app, the games, and if appropriate, their reasons for giving up their quit attempt early. Having indicated that their quit attempt has failed, the user is able to start a new quit attempt at any time, which will reset all their existing data, triggering a new entry survey, and so on.

All collected data is stored and uploaded to our webserver for analysis and review. For the sake of privacy, we do not collect any personally identifying information, although we do use a unique identifier so that we can track a given user across potentially many quit attempts.

### Progress

The first page of the main dashboard is titled “Progress,” and includes tracked statistics relating to the financial, health, and social outcomes of the user associated with their current quit attempt. It delivers an easily accessible snapshot of the statistics that are most relevant/important to the user at the current time (based on their current status and data collected during a commencement survey). The user can optionally explore a comprehensive list of every tracked statistic. These progress bars provide meaningful feedback to the user as well as educating them about the benefits of quitting, and have proven to be a valuable feature in a range of successful smoking cessation apps [20,24].

The progress page also includes an achievements section, with a range of predefined goals for the user to strive toward, as well as the ability to set their own financial savings goals to target. The progress page displays the next several achievements that the user is close to achieving, but the user can click on the tile to review the full list of achievements, including achievements they have already obtained. This style of achievement system is a proven staple across the games industry, and is a mainstay in any “gamified” apps. The achievements system ensures that the user always has both short and longer term goals in sight, as well as having a set of accomplishments they can reflect upon to remind themselves how far they’ve come [20].

### Support

**Figure 3:**
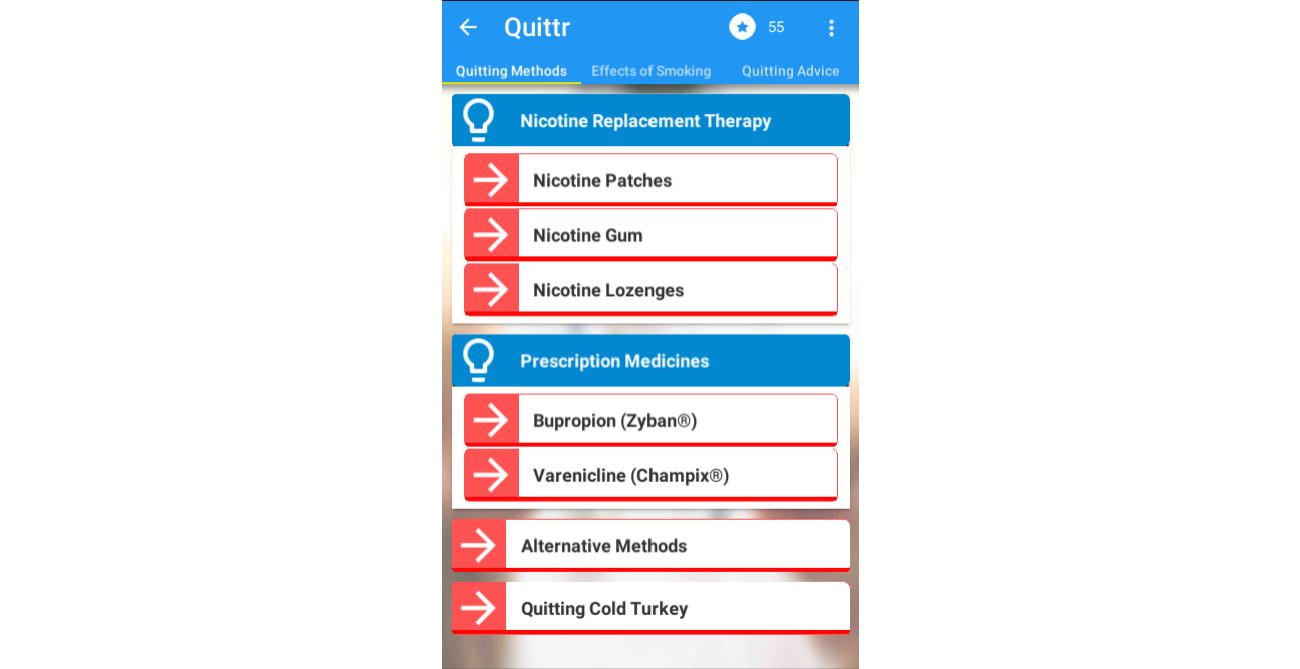
The Information Toolbox.

The support page includes a range of helpful information and support, including a button, which simply dials the local Quit Support hotline, and the “Quick Tips” button that displays helpful tips about how to use the app. Perhaps the most interesting feature of this section is the “Information Toolbox” (Figure 3).

The Information Toolbox includes a wide variety of educational material, which can support the user on their quit attempt. This includes information about the various therapies and treatment plans available, as well as information on cravings, coping strategies, the benefits of quitting, and the consequences of smoking. The information is neatly structured into ‘bite-sized chunks’ to make it easy for the user [22]. After reading a section the user is challenged by a multiple choice quiz that asks one of several basic questions that relate to the material they just read.

We have designed the content in this section to be readily customized and expanded upon, with updates being pulled from our webserver as required whenever Internet connectivity is available. The content is also tagged with a set of metadata that enables it to be tailored to the user based on the data collected during the entry survey and the state of their current quit attempt (ie, content about smoking during pregnancy would be more relevant to pregnant women than to men, and content regarding cardiovascular disease would be more important to older smokers).

### Games

**Figure 4:**
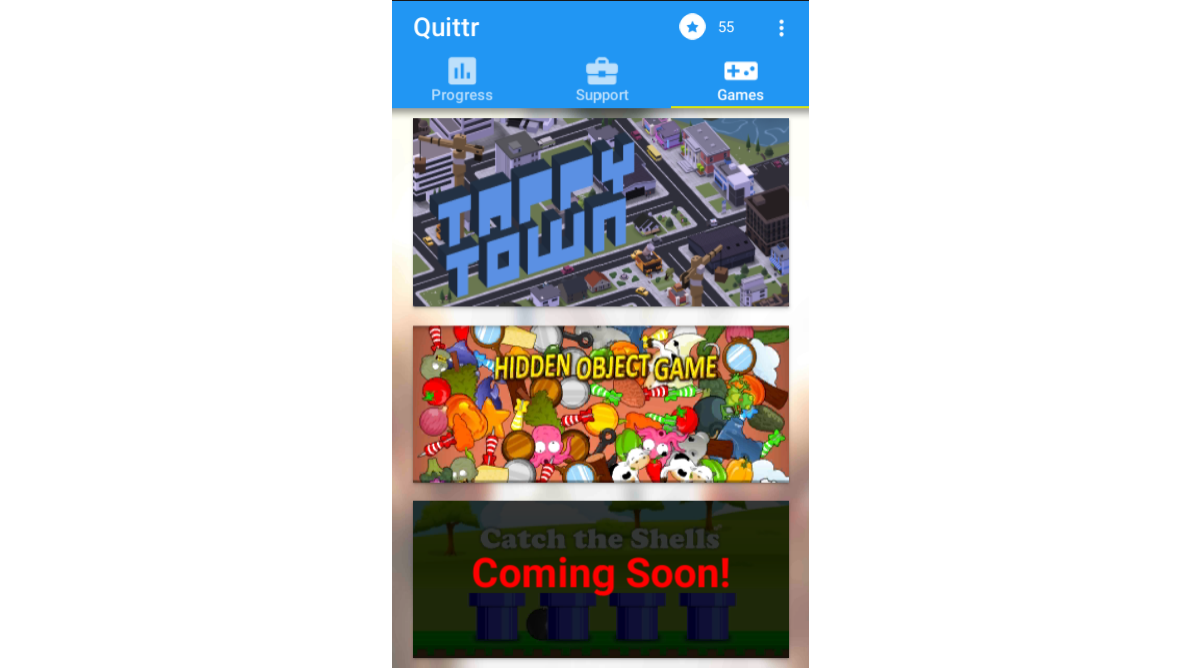
The games currently available in Quittr.

The games page lists a range of games that the user can engage with (Figure 4). The games are split into 2 categories, those that are intended purely as a distraction from cravings, and those that are used as an incentive to engage with the broader app. Currently, 2 games are available, with plans to include more. By providing a variety of games that will appeal to different users, we can improve the likelihood that a given user will be able to find a game they enjoy playing, and that they find it to be an effective distraction.

#### Distraction

The distraction games are simple minigames that can be played in a stand-alone 1- to 5-minute session. They are designed to demand mental focus and both hands, so as to provide a meaningful and effective distraction or an alternative from the act of smoking [5,16,22].

There is currently only one distraction minigame included in Quittr. This is a hidden object style game where the user must search for and tap particular objects in an increasingly cluttered environment, and under time pressure. There are also 2 additional games being prepared for inclusion: a fruit-ninja style game where the user must swipe across missiles, which are launched while avoiding bombs; and an endless-runner style game where the user must navigate a plane through a series of winding caverns without crashing while collecting coins and powerups.

#### Incentivization

Incentivization games are designed to be played incrementally over the 28-day duration of the quit attempt. These are games that are designed to involve longer term goal setting and planning, as well as frequent monitoring/check-ins by the user. Most importantly, they make use of a premium currency system, which we have called QuitCoins. This system will be familiar to those who have played free-to-play mobile and Facebook games, wherein the user can spend a premium currency (traditionally obtained by spending real money or engaging with video ads) to obtain bonuses in the game. In this case, instead of purchasing QuitCoins using real money, the user is rewarded with QuitCoins in return for engaging with the smoking cessation content of the broader app in a meaningful way (see Textbox 1). This is the key innovation that our approach adds, distinguishing it from earlier attempts in this space.

Currently, we have only created 1 incentivization game. It is a “city builder” style game, which we have called “Tappy Town.” In Tappy Town, the user has an empty plot of land to fill and a large range of structures they can build to improve that land. As the user grows their town the town’s economy will also grow, enabling them to afford increasingly grand and powerful structures. The town will passively collect resources over time, but users are rewarded for frequent check-ins by the intermittent availability of significant bonus resources that require manual collection by tapping the associated buildings. This ensures that every 1 to 2 hours (corresponding to when we might expect the next major craving to hit) the user will have something productive to do in their town, and also provides an additional mechanism by which the user can distract themselves from their cravings.

**The ways the user can earn QuitCoins.**

- Completing the entry survey
- Opening the app
- Reporting how many cigarettes they’ve smoked
- Reading through their dashboard statistics
- Reading support content
- Successfully completing quizzes
- Playing a distraction minigame
- Earning achievements
- Setting up and achieving personal goals
- Completing the exit survey

**Figure 5:**
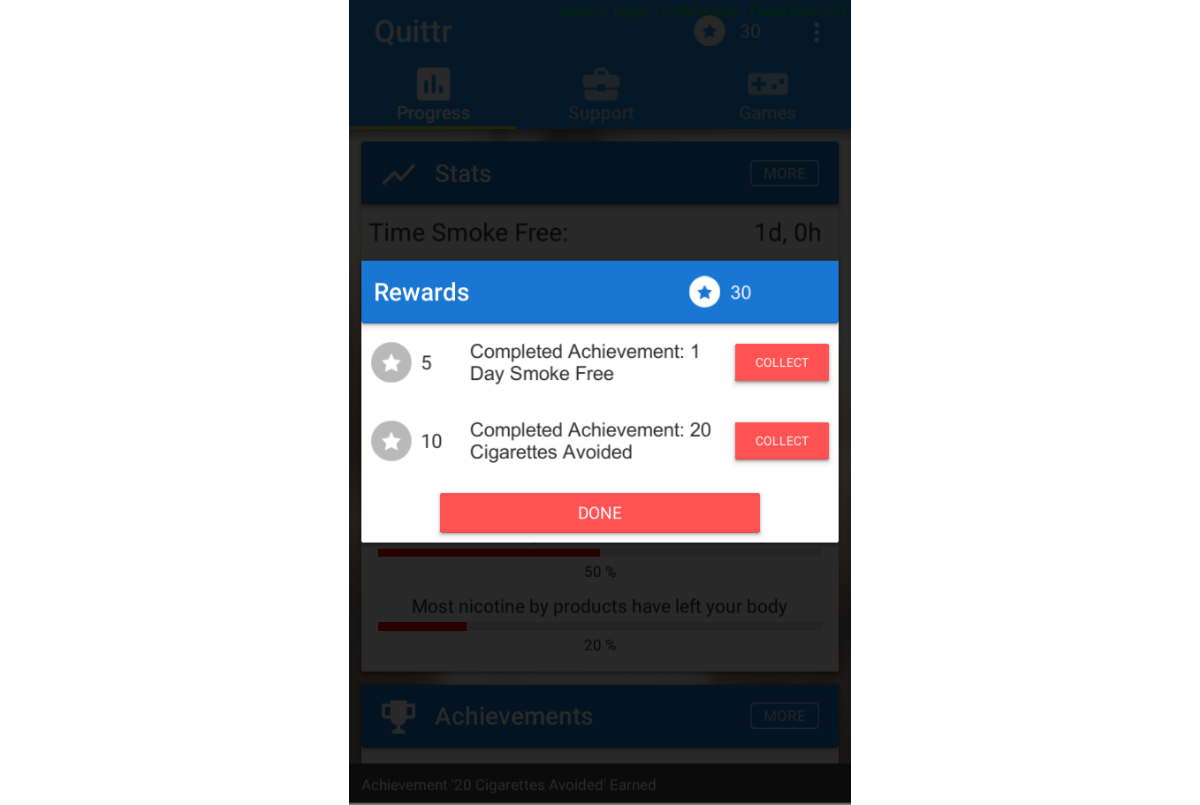
Collecting QuitCoin rewards.

However, the QuitCoin rewards for a given piece of content can only be earned periodically. For example, after reading a particular piece of advice the user might be rewarded with a QuitCoin, but they cannot reread the same piece of advice to earn another QuitCoin; they must wait a day before they can earn rewards from reading that particular piece of advice again. QuitCoins are awarded immediately after performing the related action, and this is made visible to the user by an animation and sound-effect that plays in the top right corner of the dashboard. When the user taps the QuitCoin icon they are presented with a list of all the QuitCoin rewards they have been awarded recently. This ensures that the user understands clearly why they earned the reward (Figure 5).

Having earned QuitCoins, the user can redeem these coins in Tappy Town (or any other incentivization game we might add at a later date). The highest performing and most visually impressive structures can only be purchased using QuitCoins. The intention is that the user will become hooked on the reward mechanisms on display in Tappy Town, and will develop a sense of ownership of the town they are gradually building. This will inspire them to engage more with the various contents in the Quittr app so that they can afford to build these impressive showcase structures in their town. These mechanisms are readily apparent to the user, who is regularly notified that they are earning QuitCoins through natural interactions with Quittr, and who are then encouraged to go to Tappy Town to redeem them.

## Results

At the time of publication Quittr is largely complete, with only content revision still required. It has been in closed (invite only) beta testing using Apple TestFlight and on the Android Play Store since August 9, 2016, and has at the time of writing (October 4, 2016) been installed by 7 users on iOS and 11 users on Android. The feedback received during this beta testing period has resulted in 6 iterative beta releases during this period to date. At this time, we are confident that Quittr is stable and all features are working as intended. The open beta test is planned to become available for 2017, pending ethics board approval.

## Discussion

### Further Work

It has yet to be determined whether the innovative game systems we have added into the traditional mHealth smoking cessation recipe provide the additional value we are hoping for in terms of engagement, retention, and ultimately cessation rates. To test the impact of the gaming elements, we are currently planning a research project using a randomized controlled trial design. Users will be randomized into 1 of 2 groups, either the control group that gets access to a “baseline” version of the game without the incentivization game features and the QuitCoin premium currency system, and the intervention group, which receives a copy of the game with the full feature set.

By comparing the results of these 2 groups, we will be able to determine whether the additional incentivization game features we have developed offer any value in terms of the key metrics of engagement–how much content users are consuming; retention–how many days that users are persisting with using the app; and smoking cessation rates–what proportion of users successfully abstain from smoking for given period of time (based on user-reported data and biochemically verified).

Going beyond this, we would ultimately like to compare our approach against a usual care scenario, to see how it compares more directly against current practice. We would also like to do a more specific evaluation in the younger demographic because we believe that our approach may be particularly useful for younger smokers, who are perhaps more willing to engage with a mobile phone–based intervention, and with game-based approaches in general.

### Closing Thoughts

If this model proves successful there is a broad range of potential applications for this approach. By using the virtual currency approach in the way we have done here, we remove the need for the game to comprehensively integrate the healthy activity as part of its actual play mechanics. This opens up the potential for a wide variety of health problems to be tackled through games where no obvious play mechanic presents itself. This may be of benefit in areas such as managing chronic conditions (diabetes, heart disease, etc), treating substance abuse (alcohol, illicit drugs, etc), diet and exercise, eating disorders (anorexia, bulimia, and binge eating), and various phobias.
